# Contribution of Zinc Solubilizing Bacteria in Growth Promotion and Zinc Content of Wheat

**DOI:** 10.3389/fmicb.2017.02593

**Published:** 2017-12-21

**Authors:** Sana Kamran, Izzah Shahid, Deeba N. Baig, Muhammad Rizwan, Kauser A. Malik, Samina Mehnaz

**Affiliations:** ^1^Department of Biological Sciences, Forman Christian College (A Chartered University), Lahore, Pakistan; ^2^Department of Chemistry, Government College Township, Lahore, Pakistan

**Keywords:** PGPR, zinc solubilization, atomic absorption spectroscopy, zinc quantification, grain zinc content, exopolysaccharides

## Abstract

Zinc is an imperative micronutrient required for optimum plant growth. Zinc solubilizing bacteria are potential alternatives for zinc supplementation and convert applied inorganic zinc to available forms. This study was conducted to screen zinc solubilizing rhizobacteria isolated from wheat and sugarcane, and to analyze their effect on wheat growth and development. Fourteen exo-polysaccharides producing bacterial isolates of wheat were identified and characterized biochemically as well as on the basis of 16S rRNA gene sequences. Along these, 10 identified sugarcane isolates were also screened for zinc solubilizing ability on five different insoluble zinc sources. Out of 24, five strains, i.e., EPS 1 (*Pseudomonas fragi)*, EPS 6 (*Pantoea dispersa)*, EPS 13 (*Pantoea agglomerans)*, PBS 2 (*E. cloacae)* and LHRW1 (*Rhizobium* sp.) were selected (based on their zinc solubilizing and PGP activities) for pot scale plant experiments. ZnCO_3_ was used as zinc source and wheat seedlings were inoculated with these five strains, individually, to assess their effect on plant growth and development. The effect on plants was analyzed based on growth parameters and quantifying zinc content of shoot, root and grains using atomic absorption spectroscopy. Plant experiment was performed in two sets. For first set of plant experiments (harvested after 1 month), maximum shoot and root dry weights and shoot lengths were noted for the plants inoculated with *Rhizobium* sp. (LHRW1) while *E. cloacae* (PBS 2) increased both shoot and root lengths. Highest zinc content was found in shoots of *E. cloacae* (PBS 2) and in roots of *P. agglomerans* (EPS 13) followed by zinc supplemented control. For second set of plant experiment, when plants were harvested after three months, *Pantoea dispersa* (EPS 6), *P. agglomerans* (EPS 13) and *E. cloacae* (PBS 2) significantly increased shoot dry weights. However, significant increase in root dry weights and maximum zinc content was recorded for *Pseudomonas fragi* (EPS 1) inoculated plants, isolated from wheat rhizosphere. While maximum zinc content for roots was quantified in the control plants indicating the plant's inability to transport zinc to grains, supporting accelerated bioavailability of zinc to plant grains with zinc solubilizing rhizobacteria.

## Introduction

Despite the growing industrial and technological advancement, agriculture is still the most significant sector of Pakistan, contributing one-fifth of the total GDP. Wheat is considered to be one of the chief food crops in Pakistan, contributing 10.3% to the agricultural sector and having annual production of 25.3 million tons, calculated in 2013–2014 (Mirza et al., 2015). Zinc, one of the domineering micronutrients, is required in small amount for the proper growth and development of living organisms (Hafeez et al., 2013). In plants, specifically, it is involved in carbohydrate metabolism (Alloway, 2008), auxin metabolism (Alloway, 2004) and acts as a significant anti-oxidant. Zn-finger transcription factors play an important role in the normal development of floral tissues, flowering, fertilization and fruiting (Epstein and Bloom, 2005). Zinc deficiency in plants leads to retarded shoot growth, chlorosis, reduced leaf size (Alloway, 2004), susceptibility to heat, light and fungal infections, as well as affects grain yield, pollen formation, root development, water uptake and transport (Tavallali et al., 2010). Zinc deficiency in wheat leads to yellowing of leaves and stunted growth. Consuming zinc deficient wheat can lead to zinc deficiency in humans as well.

Plants can uptake zinc as divalent cation (Kabata-Pendias and Pendias, 2001) but only a very minor portion of total zinc is present in soil solution as soluble form. Rest of the zinc is in the form of insoluble complexes and minerals (Alloway, 2008). Due to unavailability of zinc in soil, zinc deficiency occurs which is one of the most widespread micronutrient deficiency. To alleviate zinc deficiency, various methods have been applied since long. Zinc fertilizers in the form of zinc sulfate (White and Broadly, 2005) or Zn-EDTA (Karak et al., 2005) have been used, but their usage puts an economical and environmental pressure and these are transformed into insoluble complex forms within 7 days of fertilizer application (Rattan and Shukla, 1991). Regular crop rotation and intercropping has been used in various areas (Gunes et al., 2007; Zuo and Zhang, 2009) to promote zinc uptake by plants. Other methods include conventional breeding (Cakmak et al., 2010), transgenic approaches and genetic engineering (Gustin et al., 2009; Mhatre et al., 2011; Tan et al., 2015). However, these approaches are expensive, laborious and slower. A better alternative to all these approaches is the use of zinc solubilizing rhizobacteria.

Plant growth promoting rhizobacteria (PGPR) are soil borne bacteria that colonize the rhizosphere, multiply and compete with other bacteria to promote plant growth (Kloepper and Okon, 1994). PGPR promote plant growth either by solubilizing and assisting nutrient acquisition or by releasing phytohormones or biocontrol agents to protect plant from various pathogens (Glick, 2012). Various PGPR have found to be effective zinc solubilizers. These bacteria improve the plant growth and development by colonizing the rhizosphere and by solubilizing complex zinc compounds into simpler ones, thus making zinc available to the plants.

Zinc solubilizing microorganisms solubilize zinc through various mechanisms, one of which is acidification. These microbes produce organic acids in soil which sequester the zinc cations and decrease the pH of the nearby soil (Alexander, 1997). Moreover, the anions can also chelate zinc and enhance zinc solubility (Jones and Darrah, 1994). Other mechanisms possibly involved in zinc solubilization include production of *siderophores* (Saravanan et al., 2011) and proton, oxido-reductive systems on cell membranes and chelated ligands (Wakatsuki, 1995; Chang et al., 2005). Various PGPR have shown enhanced growth and zinc content when inoculated in plants. These include *Pseudomonas, Rhizobium* strains (Deepak et al., 2013; Naz et al., 2016), *Bacillus aryabhattai* (Ramesh et al., 2014), *Bacillus* sp. (Hussain et al., 2015), and *Azospirillum*. Bacterial strains that have been reported to show zinc solubilization on lab scale include *Pseudomonas aeruginosa* (Fasim et al., 2002), *Gluconacetobacter diazotrophicus* (Saravanan et al., 2007), *Bacillus* sp., *Pseudomonas striata, Pseudomonas fluorescence, Burkholderia cenocepacia* (Pawar et al., 2015), *Serratia liquefaciens, S. marcescens*, and *Bacillus thuringiensis* (Abaid-Ullah et al., 2015). Prospective zinc solubilizing bacteria for enhanced nutrition and zinc uptake in *Zea mays* L., zinc solubilizing *Bacillus* strains that modulate growth, yield and zinc biofortification of soybean and wheat have also been characterized by researchers (Khande et al., 2017). These strains have been reported for increasing zinc content of straw and grains in soybean and wheat, enhancing food efficacy and coping with zinc deficiency. Vaid et al. (2014) have reported rice growth promotion and 42.7% increased zinc nutrition of grains when inoculated with zinc solubilizing bacteria.

Keeping in view the above facts, this study was designed to identify and characterize pre-isolated bacteria from wheat and sugarcane for plant growth promoting (PGP) abilities, zinc solubilizing ability using plate assays and to evaluate the contribution (if any), of zinc solubilizing strains on growth and zinc content of wheat plants, through pot experiments.

## Materials and methods

### Bacterial isolates

In this study, 14 un-identified exopolysaccharide (EPS) producing bacterial isolates, previously isolated from wheat (Mehnaz, unpublished) and 10 identified strains from sugarcane: PBS1, PBS2, QS2, QST-W1, LHR-Sterilized, LHST-IN-W1, LHR-W1, LHR-W2, LS1-a, and LS1-b (Mehnaz et al., 2010) were used and maintained on LB agar plates (Bertani, 1952) at 28°C. Colony morphology of these isolates was observed on LB agar and RCV-sucrose agar media (Weaver et al., 1975) by incubating at 28°C for 24–48 h.

### Biochemical and molecular characterization of bacterial isolates

Gram staining of the unidentified wheat isolates was performed using the standard procedure described by Vincent (1970) and biochemical tests were performed using bacterial miniaturized identification kits QTS-24 (DESTO Laboratories, Karachi, Pakistan) following manufacturer's instructions.

Genomic DNA of unidentified isolates was extracted using Genomic DNA purification kit (ThermoScientific^TM^ GeneJET^TM^ USA). 16S rRNA gene for each bacterial isolate was amplified in 50 μl reaction mixture containing 25 μl of PCR Dream *Taq* master mix; Taq DNA polymerase (0.05 U/μl), reaction buffer, MgCl_2_ (4 mM) and dNTPs, 0.4 mM each (Thermo Fisher Scientific, USA; Catalog Number: EP0701), 5 μl template DNA, 12 μl diH_2_O and 8 μl of 20 pmol P15 (5′-CGGGATCCAGAGTCAGAACGAACGCT-3′and P65- 5′CGGGATCCTACGGACGACTTCACCCC-3′) universal primers (Tan et al., 1997). Initial denaturation was provided at 94°C for 2 min following 35 cycles of 94°C for 1 min, 55°C for 1 min, 72°C for 3 min and final extension at 72°C for 10 min (Chun and Goodfellow, 1995). The amplified (1.5 kb) 16S rRNA gene products were analyzed using gel electrophoresis by running DNA on 1% agarose gel at 90 V for 40–50 min. and concentration was determined by Nanodrop UV spectrophotometer (ThermoScientific, USA; Model no: E4-106-50-0001-S). Gene products were purified with PCR Purification Kit (Thermo Fisher Scientific, USA), commercially sequenced (Eurofins, Germany) and sequences were BLAST (Basic Local Alignment Search Tool) for homology.

Multiple Sequence alignment of all bacterial isolates was carried out using CLUSTALW (ver. 1.83). *Planctomycete* was used as out-group to construct Phylogenetic Tree and evolutionary history was created using neighbor-joining method. Percentage of replicate trees in which associated taxa clustered together in the bootstrap test (1,000 replicates) are shown next to the branches. The evolutionary distances were calculated using the maximum composite likelihood method and were in the units of number of base substitutions per site. All positions containing gaps and missing data were eliminated from the dataset using the complete deletion option. There were a total of 1,408 positions in the final dataset. Phylogenetic analysis was conducted with MEGA4 software. These sequences were deposited in the GenBank database and accession numbers were obtained (i.e., KY848812 to KY848824 and MF150305).

### Plant growth promoting (PGP) ability assays

#### Indole-3- acetic acid (IAA) production assay

Single bacterial colonies of individual strains were inoculated in 100 ml LB broth containing 0.1% L-tryptophan as a precursor for IAA production and incubated at 120 rpm, 28°C for 7 days. Qualitative assessment was done by harvesting cells from 500 μl culture in sterile eppendorf tubes at 10,000 rpm for 10 min and adding 1 ml Salkowski's reagent (Gordon and Weber, 1950) in supernatant. Samples were kept in dark for 15–20 min to observe color change.

For quantitative assessment, cells from 7 days old, 100 ml bacterial culture grown in LB with 0.1% L-tryptophan, were harvested at 10,000 rpm for 15–20 min. The pH of the supernatant was adjusted at 2.8 using hydrochloric acid and it was extracted twice with equal volumes of ethyl acetate (Tien et al., 1979) following evaporation using rotary evaporator and was resuspended in 1 ml methanol. The samples were analyzed by high-performance liquid chromatography (HPLC) on Waters HPLC System (e2995, separations module) with 2998 photodiode-array (PDA) detector using a Nucleosil C18 column (4.6 × 250 mm, 5 μM; Macherey-Nagel, Germany). The mobile phase was a mixture of methanol/acetic acid/water (30:1:70, v/v/v) and the flow rate was adjusted at 1.2 ml /min (Rasul et al., 1998). Pure indole-3-acetic acid (Sigma) was used to prepare standard solutions.

#### Siderophore and hydrocyanic acid (HCN) production assay

For qualitative assessment of siderophore production, O-CAS method as described by Perez-Miranda et al. (2007) was used. Each bacterial culture was spot inoculated on LB agar plate and incubated at 28°C for 1–2 days. CAS medium was prepared as described by Schwyn and Neilands (1987). PIPES buffer medium and CAS dye solution were mixed and applied to 48 h grown bacterial cultures, as overlay. The plates were kept at 28°C for 20 min to 24 h.

Qualitative production of HCN was determined by streaking bacterial colonies on LB agar plates. Filter papers dipped in alkaline picrate solution (0.25% picric acid and 1.25% sodium carbonate) were placed on the lids of petri plates (Miller and Higgins, 1970), sealed with parafilm and incubated at 28°C for 4-5 days.

#### Extracellular enzyme assays

Overnight grown bacterial cultures were transferred aseptically by spot inoculation on 1% Tween-20 LB agar plates to detect lipase production (Sierra, 1957). National Botanical Research Institute's Phosphate Growth medium (NBRIP; Nautiyal, 1999) plates were used for detection of P solubilization ability by isolates. LB agar plates supplemented with 1% carboxymethyl cellulose (CMC) for cellulase production (Lin et al., 2012) and skim milk agar plates (Kumar et al., 2005), were used to detect the production of proteases. Fresh bacterial cultures were spot inoculated and the plates were incubated at 28°C for 3–4 days for lipase and protease and 14 days for phosphatase production ability. Clear zones around colonies indicated respective activity. For cellulase, plates were stained with 0.1% congo red for 15 min, following de-staining (1M NaCl for 15 min). Formation of yellowish zones around bacterial growth indicated positive results.

#### *In vitro* zinc solubilization assessment using plate assay

All bacterial strains were screened for their zinc solubilizing ability for five insoluble zinc compounds viz. zinc sulfate (ZnSO_4_), zinc oxide (ZnO), zinc chloride (ZnCl_2_), zinc phosphate Zn_3_(PO_4_)_2_ and zinc carbonate (ZnCO_3_). Overnight grown single colonies were transferred aseptically by inoculating as spot on respective zinc medium plates (Sharma et al., 2012). These plates were covered with aluminum foil and incubated in dark at 28°C for 14 days. Zinc solubilizing strains produced clear zones around colonies. The diameter of these zones was recorded.

### Plant experiments

To analyze the effect of zinc solubilizing bacteria on wheat growth and development, plant experiments were carried out. Based on zinc solubilizing ability, five strains; *Pseudomonas fragi* (EPS 1), *Pantoea dispersa* (EPS 6), *Pantoea agglomerans* (EPS 13), *Enterobacter cloacae* (PBS 2), and *Rhizobium* sp. (LHRW1) were selected for plant experiments and zinc carbonate (ZnCO_3_) was used as zinc source. In total, seven treatments with 10 replicates each, were designed as follows: Control (without zinc source + without bacteria), Control+ZnCO_3_ (without bacteria), ZnCO_3_ + EPS 1, ZnCO_3_ + EPS 6, ZnCO_3_ + EPS 13, ZnCO_3_ + PBS 2 and ZnCO_3_ + LHRW1. Seventy plastic pots of 11.5 cm diameter and 10.5 cm height were labeled and 500 g autoclaved oven-dried sand was added per pot. One percent ZnCO_3_, i.e., 5 g ZnCO_3_ per 500 g sand was added in each of the 60 pots excluding 10 pots of the control treatment. For seed sterilization, 100 seeds of wheat variety, i.e., Faisalabad-2008 were soaked in 100 ml of 10% bleach solution for 15 min, and four successive washes of 10 min each were given using 100 ml autoclaved water (Cheng et al., 1997). Using sterile forceps the seeds were carefully transferred to 1% water-agar plates and sealed plates were incubated at 28°C for 3 days to allow germination.

Zinc solubilizing isolates were individually inoculated in 60 ml LB broth and grown in shaking incubator at 120 rpm, 28°C. After 24 h, the optical density of each strain was adjusted at 1.0 at the wavelength of 600 nm. Cells were harvested from 50 ml culture at 6,000 rpm for 20 min and resuspended in 0.85% saline, containing 10^8^ cells/ml for inoculum. Fifty milliliter sterile full strength, nitrogen containing Hoagland's solution (Hoagland and Arnon, 1950) was added in each pot. Using sterilized forceps, 3-days old seedlings were transferred in pots and 1 ml bacterial inoculum was given to each respective pot. Pots were placed in a climate room and arranged in a Randomized Complete Block Design (RCBD). The temperature in climate room was maintained at 20 ± 2°C; with light source of 6,000 ± 500 FLUX and light period of 10 ± 1 h. To provide moisture, plants were watered at alternate days using autoclaved distilled water. Two sets of experiments were carried out. For first set, Plants were harvested after 1 month. This experiment was repeated twice. However, for second set, plants were harvested after 3 months (at grain level). Roots were washed using tap water and separated from shoots. Lengths, fresh, and dry weights of all samples including shoots and roots were measured. Data was statistically analyzed using the Statistical Package for the Social Sciences (SPSS) software (IBM Statistics 23.0).

#### Analysis of zinc content using atomic absorption spectroscopy

Roots, shoots and grains of three plant samples per treatment were analyzed for zinc estimation. 0.1 g plant material was digested in di-acid mixture containing 25 ml concentrated nitric acid and 10 ml concentrated sulphuric acid. The mixture was heated at 70°C until complete digestion and diluted using 50 ml distilled water. Extract was filtered through a Whatman's filter paper (Jepkoech et al., 2013) and samples were analyzed for total zinc content using atomic absorption spectrophotometer (Varian AA 240 F.S., USA).

## Results

### Biochemical and molecular characterization of bacterial isolates

**Figure 1:**
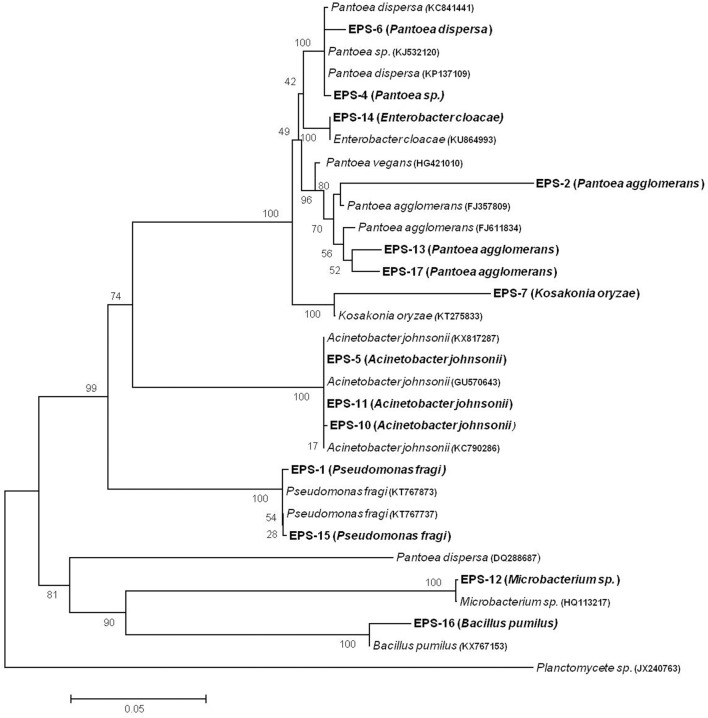
Neighbor-joining tree of 16S rRNA gene sequences of wheat EPS strains. Sequences of closest members were obtained from database and accession numbers are mentioned. There were total of 1,408 positions in final dataset. The percentage of replicate trees in which the associated taxa clustered together in the bootstrap test (1,000 replicates) is shown next to the branches. The evolutionary distances were computed using the maximum composite likelihood method and are in units of number of base substitutions per site.

Based on gram staining, cell morphology and biochemical tests, wheat isolates were characterized according to Bergey's manual of determinative bacteriology (Table 1). Based on 16S rRNA sequence analysis, two isolates were identified as *Pseudomonas fragi* (EPS 1, EPS 15), five isolates as different species of *Pantoea*, i.e., *P. dispersa* (EPS 6), *Pantoea sp*. (EPS 4) and *P. agglomerans* (EPS 2, EPS 17, EPS 13), respectively; three isolates as *Acinetobacter johnsonii* (EPS 5, EPS 10, EPS 11), one as *Kosakonia oryzae* (EPS 7), one as *Enterobacter cloacae* (EPS 14), EPS 12 as *Microbacterium sp*., and one as *Bacillus pumilus* (EPS 16). Phylogenic tree is shown in Figure 1. Sequence lengths, accession numbers and percentage homology of each strain is summarized in Table 2.

**Table 1:** Biochemical Characterization of EPS isolates using QTS-24 identification kits and Bergey's Manual of Identification.

**Sr. No**.	**1**	**2**	**3**	**4**	**5**	**6**	**7**	**8**	**9**	**10**	**11**	**12**	**13**	**14**	**15**	**16**	**17**	**18**	**19**	**20**	**21**	**Identification Bergey's Manual**
**Test/Bacterial Isolates**	**ONPG**	**CIT**	**MALO**	**ADH**	**ODC**	**H_2_S**	**UREA**	**VP**	**GEL**	**NO_3_/N_2_**	**MR**	**MOT**	**OX**	**GLU**	**Suc**	**RHAM**	**SORB**	**MEL**	**RAF**	**FRUC**	**MANS**	
EPS 1	−	+	+	+	+	+	+	+	−	−/−	+	+	+	+	−	−	−	−	−	−	−	*Pseudomonas fragi*
EPS 2	+	−	+	−	−	−	−	+	−	+/+	+	+	−	+	+	−	−	+	−	w	+	*Pantoea* sp.
EPS 4	+	−	+	−	−	−	−	−	+	−/−	−	−	−	w	W	−	−	+	+	+	−	*Pantoea* sp.
EPS 5	−	−	−	−	−	+	−	+	−	+/−	−	−	+	+	+	+	+	+	−	−	−	*Acinetobacter* sp.
EPS 6	+	−	−	−	−	−	−	+	−	−/−	+	+	+	+	+	+	−	+	−	+	+	*Pantoea dispersa*
EPS 7	+	−	+	−	−	+	−	+	−	+/−	+	+	+	w	+	+	+	−	−	+	W	*Kosakonia* sp.
EPS 10	−	+	+	+	+	+	+	−	−	+/−	+	−	+	+	+	+	+	+	+	+	−	*Acinetobacter johnsonii*
EPS 11	+	−	+	−	−	−	−	−	+	+/−	−	+	−	+	+	W	+	w	−	−	−	*Acinetobacter* sp.
EPS 12	−	−	+	−	−	−	−	+	−	−/−	−	−	−	−	+	−	+	+	−	+	−	*Microbacterium* sp.
EPS 13	+	−	+	−	−	−	−	+	−	−/−	+	+	+	+	−	W	−	+	−	+	+	*Pantoea vagans*
EPS 14	+	+	−	+	−	−	−	+	−	−/−	+	+	+	+	+	+	+	+	+	+	+	*Enterobacter* sp.
EPS 15	−	−	−	+	−	+	−	+	−	+/−	+	+	+	−	+	−	−	+	−	+	−	*Pseudomonas fragi*
EPS 16	−	−	−	−	−	−	−	+	+	−/−	−	+	+	+	+	−	+	+	−	−	−	*Bacillus* sp.
EPS 17	+	−	+	−	−	−	−	+	+	+/−	+	+	−	+	+	+	+	+	+	w	+	*Pantoea agglomerans*

*(+): activity, (−): no activity, (w): weak activity*.

*Symbols: ONPG, Ortho-Nitrophenyl-β-galactosidase; CIT, sodium citrate; MALO, malonate; ADH, arginine dihydrolase; ODC, ornithine decarboxylase; H_2_S, hydrogen sulfide; UREA, urease; VP, voges-proskauer; GEL, gelatin hydrolysis; NO_3_, nitrate reduction; N_2_, nitrogen gas production; GLU, glucose; SUC, sucrose; RHAM, rhamnose; SORB, sorbitol; MEL, melibiose; RAF, raffinose; FRUC, fructose; MANS, mannose*.

*All strains were positive for mannitol, (MANN) and arabinose (ARAB) and negative for inositol (INOS), indole (IND) and adonitol (ADO). All strains were found positive for maltose (MALT) except EPS-1 while only EPS-10 and EPS-15 were found positive for lysine decarboxylase (LDC) and tryptophan deaminase (TDA) respectively*.

**Table 2:** Sequence length and % homology of EPS isolates based on BLAST results.

**Sr. No**.	**Strain**	**Sequence Length**	**Query Cover (%)**	**E value**	**% Identity**	**Sequences ID**	**Accession Number**	**Identified As**
1	EPS 1	1,425	100	0.0	98	KT767873	KY848812	*Pseudomonas fragi*
2	EPS 2	1,395	100	0.0	95	FJ357809	KY848813	*Pantoea agglomerans*
3	EPS 4	1,389	100	0.0	99	KJ532120	KY848814	*Pantoea* sp.
4	EPS 5	1,139	100	0.0	99	KX817287	KY848815	*Acinetobacter johnsonii*
5	EPS 6	1,386	100	0.0	99	KP137109	KY848816	*Pantoea dispersa*
6	EPS 7	1,356	100	0.0	96	KT275833	KY848817	*Kosakonia oryzae*
7	EPS 10	1,406	100	0.0	99	KC790286	KY848818	*Acinetobacter johnsonii*
8	EPS 11	1,393	100	0.0	99	GU570643	KY848819	*Acinetobacter johnsonii*
9	EPS 12	1,434	100	0.0	97	HQ113217	KY848820	*Microbacterium* sp.
10	EPS 13	1,385	100	0.0	100	EU240963	MF150305	*Pantoea agglomerans*
11	EPS 14	1,354	100	0.0	100	KU864993	KY848821	*Enterobacter cloacae*
12	EPS 15	1,348	100	0.0	99	KT767737	KY848822	*Pseudomonas fragi*
13	EPS 16	1,390	100	0.0	99	KX767153	KY848823	*Bacillus pumilus*
14	EPS 17	1,025	100	0.0	99	FJ611834	KY848824	*Pantoea agglomerans*

### Plant growth promoting assays

#### Indole-3-acetic acid (IAA) production

For qualitative assessment of IAA, all isolates except *Bacillus pumilus* (EPS 16), displayed color change ranging from light pink to reddish, on the addition of Salkowski's reagent, indicating a positive result for IAA (Figure S1). Quantification through HPLC showed maximum indole acetic acid production by *Enterobacter cloacae* (EPS 14), i.e., 12.125 μg/ml, followed by *Pantoea agglomerans* (EPS 17) which produced 8.449 μg/ml of IAA (Figure S2). Among all isolates, *Acinetobacter johnsonii* (EPS 11) showed minimum indole acetic acid production of 0.066 μg/ml (Table 3).

**Table 3:** Plant growth promoting traits by EPS bacterial strains.

**Wheat isolates**	**Protease assay**	**Lipase assay**	**Cellulase assay**	**Phosphatase assay**	**IAA production (μg/ml)**	**Siderophore assay**
EPS 1	−	−	−	+	0.25	+
EPS 2	W	+	+	w	0.312	−
EPS 4	−	−	−	−	0.71	−
EPS 5	−	+	−	w	0.394	+
EPS 6	−	+	+	w	0.934	−
EPS 7	−	−	−	−	0.413	++
EPS 9	+	−	−	−	0.324	−
EPS 10	−	W	−	−	0.108	−
EPS 11	+	−	−	−	0.066	−
EPS 12	+	−	−	−	0.472	−
EPS 13	−	W	+	+	1.134	−
EPS 14	−	−	−	−	12.125	+
EPS 15	−	−	−	+	0.912	−
EPS 16	+	+	−	−	–	−
EPS 17	+	+	−	−	8.449	−

*−, no activity; w, weak activity; ++, very good activity*.

#### Siderophore assay and HCN production

Four isolates were siderophore positive showing color change from greenish blue to yellow. Among these four, *Kosakonia oryzae* (EPS 7) showed maximum siderophore production and produced biggest halo around bacterial colony. All isolates were found negative for HCN production as color change in filter paper was not observed (Table 3).

#### Extracellular enzyme assays

Five isolates i.e., *Pantoea agglomerans* (EPS 2), *Acinetobacter johnsonii* (EPS 5), *Pantoea dispersa* (EPS 6), *Bacillus pumilus* (EPS 16), and *Pantoea agglomerans* (EPS 17) showed whitish translucent zones around colonies indicating a positive lipase result while *Acinetobacter johnsonii* (EPS 10) and *Pantoea agglomerans* (EPS 13) showed very light zones and were weak positive for the test. For protease test, six isolates showed halo zone around the bacterial colonies and were positive for the assay while nine isolates didn't produce protease. Six isolates showed phosphate solubilization on NBRIP medium by forming a halo zone around their colonies (Figure S3). However, nine isolates did not solubilize phosphate. Among 14 bacterial isolates, three isolates i.e., *Pantoea agglomerans* (EPS 2 and EPS 13) and *Pantoea dispersa* (EPS 6) formed a light yellow/whitish zone around their colonies and hence were cellulase positive (Table 3).

#### Zinc solubilization assay

Zinc solubilization ability of the bacterial strains was evaluated by determining the zone diameter. Among all 24 wheat and sugarcane isolates, six i.e., *Pseudomonas fragi* (EPS 1), *Pantoea dispersa* (EPS 6), *Pantoea agglomerans* (EPS 13), *Enterobacter cloacae* (PBS 2 and PBS 1), and *Rhizobium* sp. (LHRW1) showed zinc solubilization zones on ZnCO_3_ medium. Maximum zone of 1.8 cm was observed for *Rhizobium* sp. (LHRW1). On ZnO medium, six isolates *Pseudomonas fragi* (EPS 1), *Pantoea dispersa* (EPS 6), *Enterobacter cloacae* (PBS 1 and PBS 2), *Rhizobium* sp. (LHRW1) and *Pantoea* sp. (LS1-b) showed solubilization. On Zn_3_(PO_4_)_2_ medium, 20 strains showed weak solubilization and four isolates, i.e., *Pantoea* sp. (EPS 4), *Acinetobacter johnsonii* (EPS 11, EPS 10) and *Klebsiella oxytoca* (LHRW2) did not show any solubilization. *Pseudomonas fragi* (EPS 15) showed maximum diameter of 1 cm on Zn_3_(PO_4_)_2_ medium. All 24 isolates did not solubilize zinc on ZnCl_2_ and ZnSO_4_ supplemented medium (Figures S4–S6, Table S1).

### Plant experiments

Among zinc sources, zinc carbonate was maximum solubilized by bacterial isolates as compared to other zinc salts, hence it was selected for zinc supplementation in plant experiments. Based on zinc carbonate solubilization ability, five strains were selected for plant experiments. These strains were also positive for indole-3-acetic acid production and phosphate solubilization. The results described below are based on average values of the ten replicates of each treatment.

#### A. plants harvested after 4 weeks (first set)

**Figure 2:**
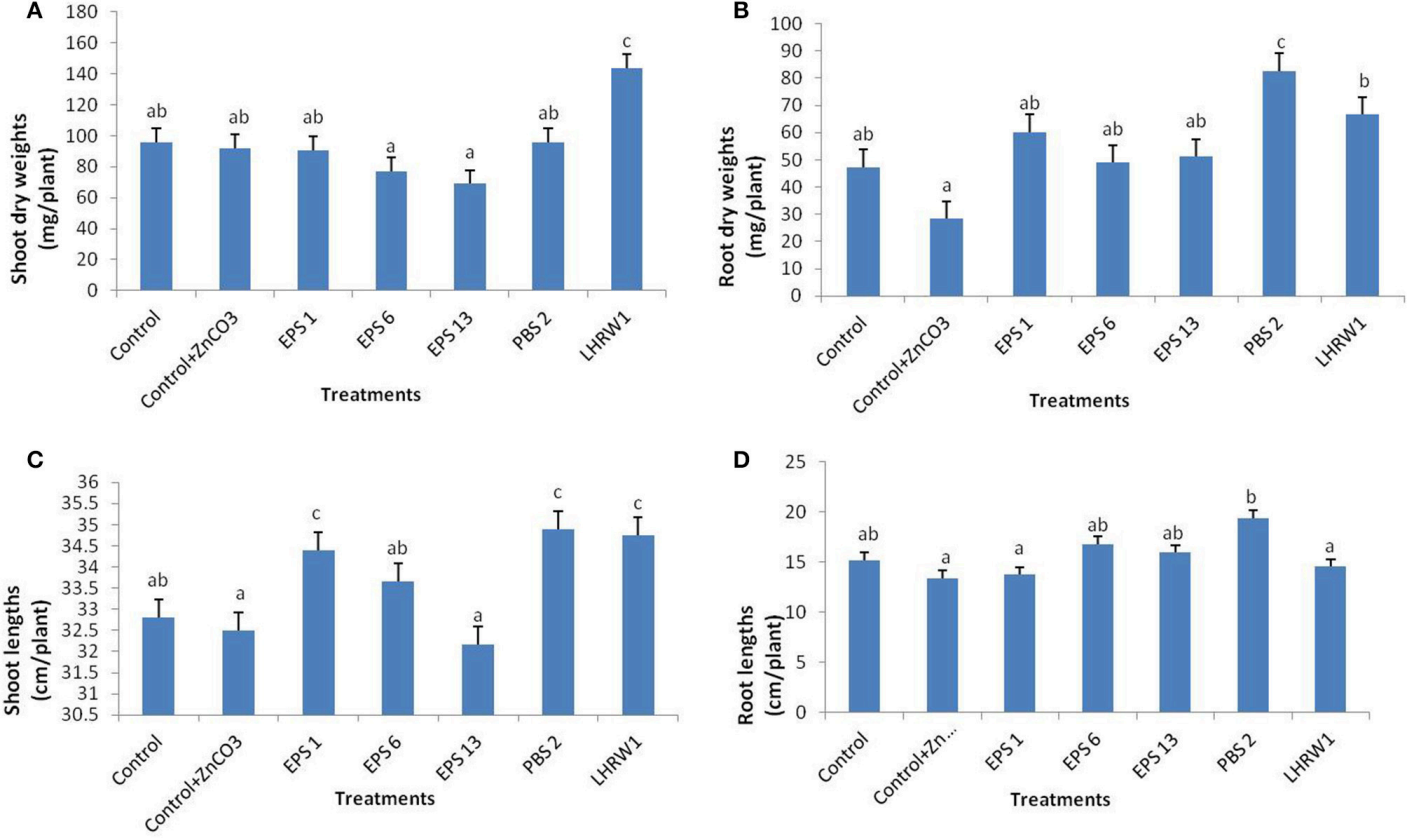
Effect of bacterial inoculation on growth parameters of plants harvested after 4 weeks (first set). **(A)** Dry weight of shoots, **(B)** Dry weight of roots, **(C)** Shoot lengths, **(D)** Root lengths in comparison with un-inoculated control. ^*^All inoculated treatments were supplemented with 1% ZnCO_3_. Alphabets (a–c) represent the significant and non-significant difference among datasets. ^a^Indicates no significant difference among dataset labeled with a on them. ^b^Shows a slight difference from (a) and same group category for dataset labeled with (b). ^c^Shows significant difference from a and b datasets and same data group labeled with c.

*Growth parameters*: The results of first set of plant experiments manifested that two of these strains; PBS 2 and LHRW1, considerably increased dry weights of roots and shoots as compared with un-inoculated plants. Significant increase in shoots dry weights was only seen for the plants inoculated with *Rhizobium* sp. (LHRW1). Plants inoculated with all other strains and un-inoculated showed almost similar shoot dry weights. No significant difference was observed in fresh root and shoot weights for inoculated and un-inoculated plants (Figure 2A).

Significantly high as well as maximum dry root weight was recorded for the plants inoculated with *Enterobacter cloacae* (PBS 2) and it was followed by *Rhizobium* sp. (LHRW1) inoculated plants. Non-significant increase was found in dry root weights of plants inoculated with all other strains as compared with un-inoculated control plants (Figure 2B, Figures S7, S8).

*Enterobacter cloacae* (PBS 2) also significantly contributed to increase lengths of roots and shoots while *Pseudomonas fragi* (EPS 1) and *Rhizobium* sp. (LHRW1) increased lengths of shoots. The maximum shoot length was recorded for *Enterobacter cloacae* (PBS 2) inoculated plants; *Pseudomonas fragi* (EPS 1) and *Rhizobium* sp. (LHRW1) followed it with minor difference. No other strain was observed to significantly increase root and shoot lengths with respect to un-inoculated controls (Figures 2C,D).

**Figure 3:**
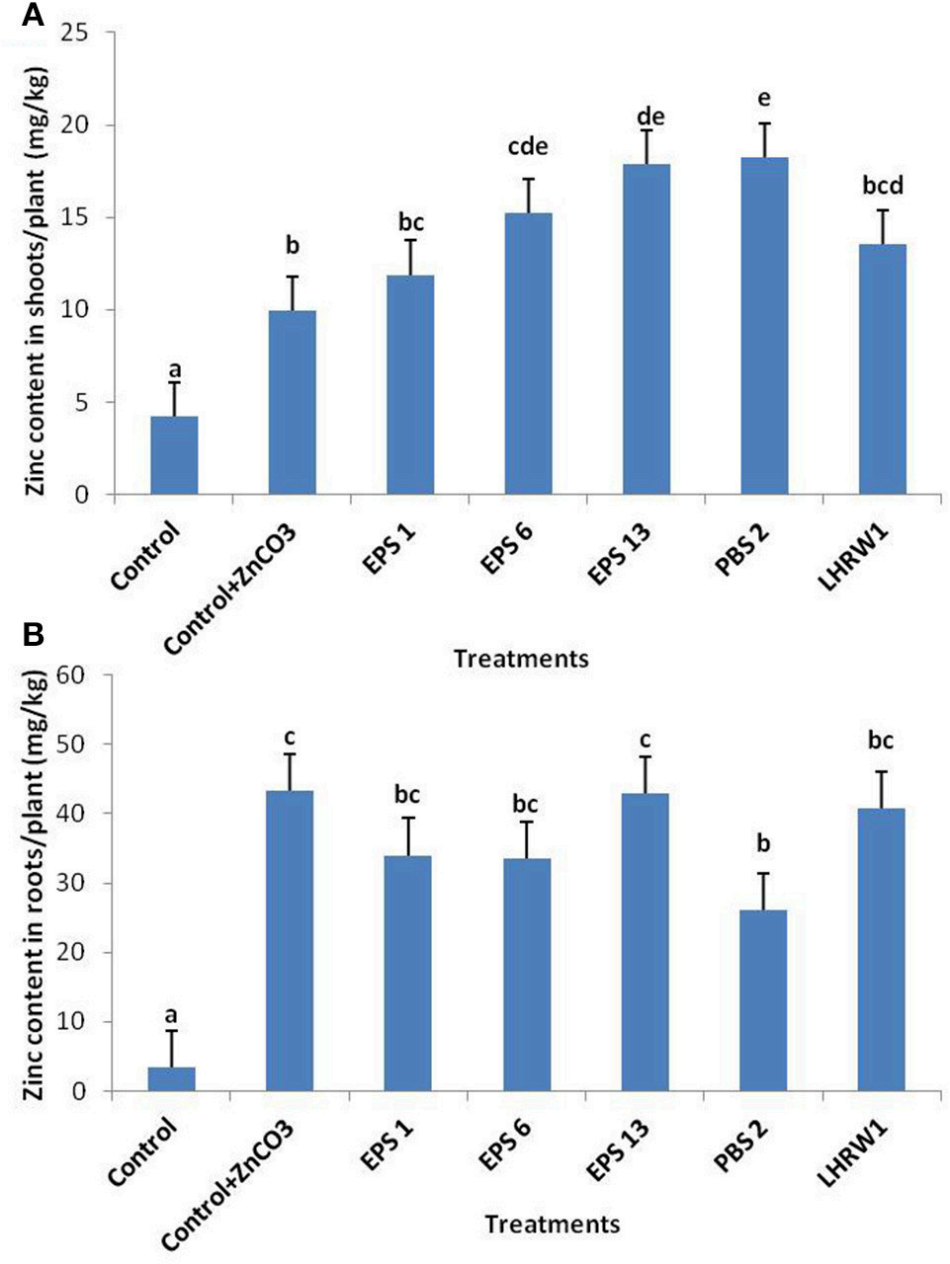
Estimation of Zinc content in **(A)** shoots and **(B)** roots of inoculated and un-inoculated plants, harvested after 4 weeks. Small letters (a–e) represent the significance and non-significance of data. All bars with same alphabet on them indicate the same group and non-significance with each other. While bars with different alphabets indicate significant difference with other groups.

*Zinc content*: Significant increase was found in zinc content of shoots and roots of inoculated plants as compared to control. The zinc content of control shoots was found to be 4.25 mg/kg. Maximum zinc content, i.e., 18.25 mg/kg was found in shoots of *Enterobacter cloacae* (PBS 2) inoculated plants, followed by *Pantoea agglomerans* (EPS 13) which showed zinc content of 17.85 mg/kg. Zinc content in shoots of other treatments ranged from ~ 12 to 15 mg/kg for inoculated plants and ~ 10 mg/kg for un-inoculated plants provided with ZnCO_3_ (Figure 3A).

Control plants that were not given any zinc also showed zinc presence in roots and shoots; 4.25 mg/kg of zinc in shoots and 3.74 mg/kg in roots. This zinc can be correlated with the amount of zinc provided to the plants in Hoagland's solution that contains 0.05 mg/l zinc. Zinc content of the shoots of control plants supplemented with ZnCO_3_ was also higher that of the control plants. This higher amount of zinc in ZnCO_3_ supplemented control is actually associated with the ability of the plant itself to mobilize available nutrients and use them for their growth.

The zinc content of roots of inoculated plants showed even more significant increase as compared to control plants. *Pantoea agglomerans* (EPS 13) showed maximum zinc content of 42.96 mg/kg in roots, followed by *Rhizobium* sp. (LHRW1) inoculated plants which showed zinc content of 40.77 mg/kg. Zinc content in roots of other inoculated treatments ranged from 26 to 34 mg/kg. However, difference was non-significant as compared to roots of un-inoculated plants provided with ZnCO_3_ (Figure 3B). Analysis of zinc content of roots and shoots demonstrate that plants can take up available zinc to their roots but its transport to the shoots can be facilitated by inoculating the plants with PGPR that can also colonize inside the plants as endophytes and make zinc bio-available to the plants.

#### B: plants harvested after 3 months (second set)

**Figure 4:**
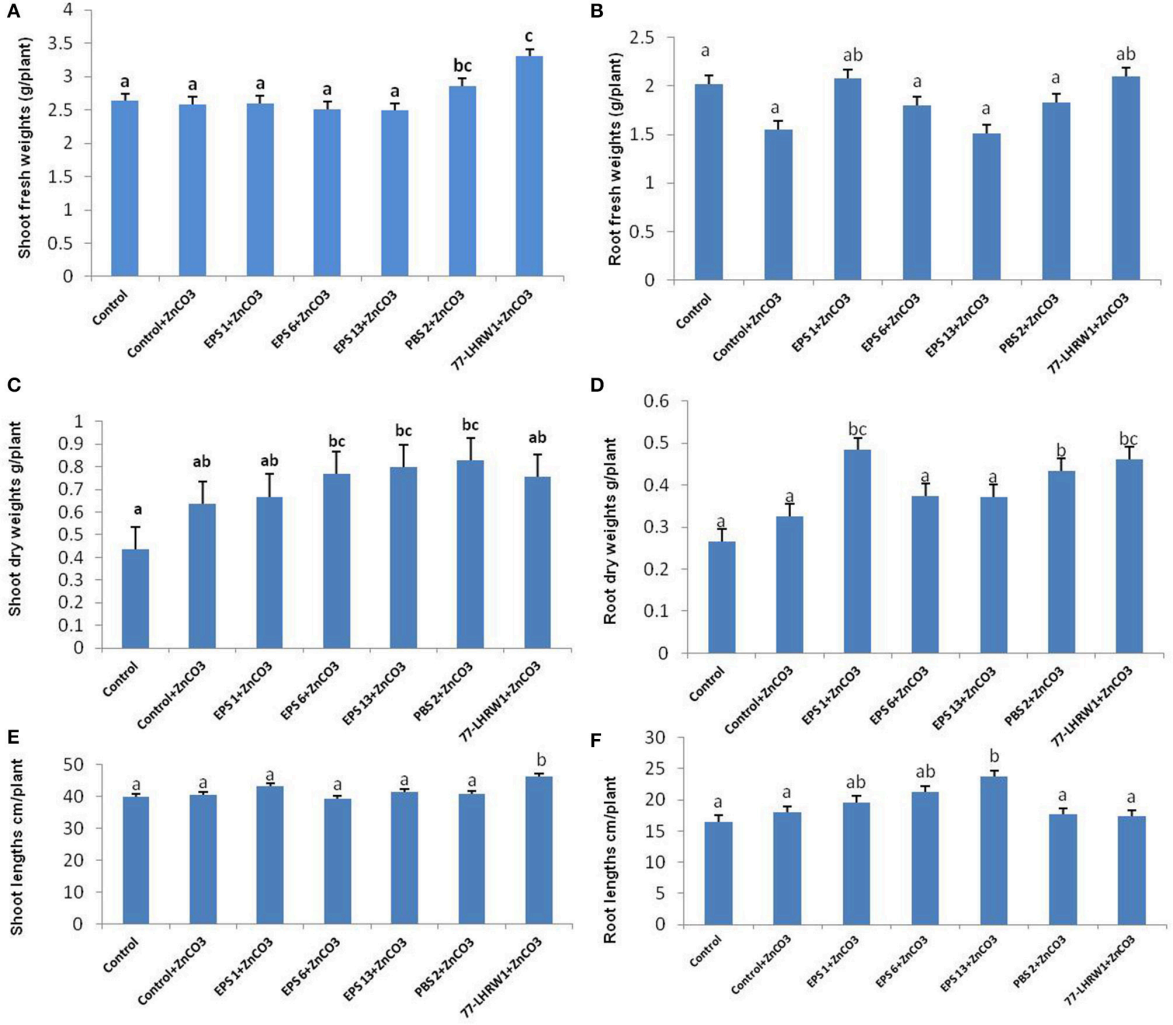
Effect of bacterial inoculation on growth parameters of plants harvested after 3 months (second set). **(A)** Fresh weight of shoots, **(B)** Fresh weight of roots, **(C)** Dry weight of shoots, **(D)** Dry weights of roots, **(E)** Shoot lengths, and **(F)** Root length. Alphabets (a–c) represent the significant and non-significant difference among datasets. ^a^Indicates no significant difference among dataset labeled with a on them. ^b^Shows a slight difference from (a) and same group category for dataset labeled with (b). ^c^Shows significant difference from a and b datasets and same data group labeled with c.

*Growth parameters*: Plants were harvested after 3 months and data for shoots and roots weights and lengths was recorded. Highest fresh weights for shoots were recorded for plants inoculated with *Rhizobium* sp. (LHRW1) followed by *E. cloacae* (PBS 2). All other strains showed insignificant difference in shoot fresh weights when compared with un-inoculated controls (Figure 4A). None of the strains showed significant increase in fresh weights of roots when compared with control plants (Figure 4B).

Most of these strains significantly increased dry weights of roots and shoots as compared with un-inoculated controls. Significant increase in shoots dry weights was seen for the plants inoculated with strains *P. dispersa* (EPS 6), *P. agglomerans* (EPS 13) and *E. cloacae* (PBS 2). Maximum dry shoot weights were recorded for the plants inoculated with *E. cloacae* (PBS 2). Plants inoculated with all other strains and un-inoculated showed almost similar shoot dry weights (Figure 4C).

*Pseudomonas fragi* (EPS 1), *E. cloacae* (PBS 2) and *Rhizobium* sp. (LHRW1) also significantly increased root dry weights as compared to controls. Plants inoculated with *Pseudomonas fragi* (EPS 1) showed highest dry root weights followed by *Rhizobium* sp. (LHRW1) and *E. cloacae* (PBS 2) when compared with un-inoculated plants. *Rhizobium* sp. (LHRW1) and *E. cloacae* (PBS 2) also significantly increased dry weights of roots in plants harvested after 4 weeks. (Figure 4D).

*Rhizobium* sp. (LHRW1) significantly increased shoot lengths while all other strains showed insignificant increase with respect to un-inoculated ones (Figure 4E). *Rhizobium* sp. (LHRW1) plants showed similar results for plants harvested after 4 weeks. *P. agglomerans* (EPS 13) inoculated plants showed maximum increase in root length. Unlike previous experiment, increase in root length of *E. cloacae* (PBS 2) inoculated plants was insignificant (Figure 4F).

**Figure 5:**
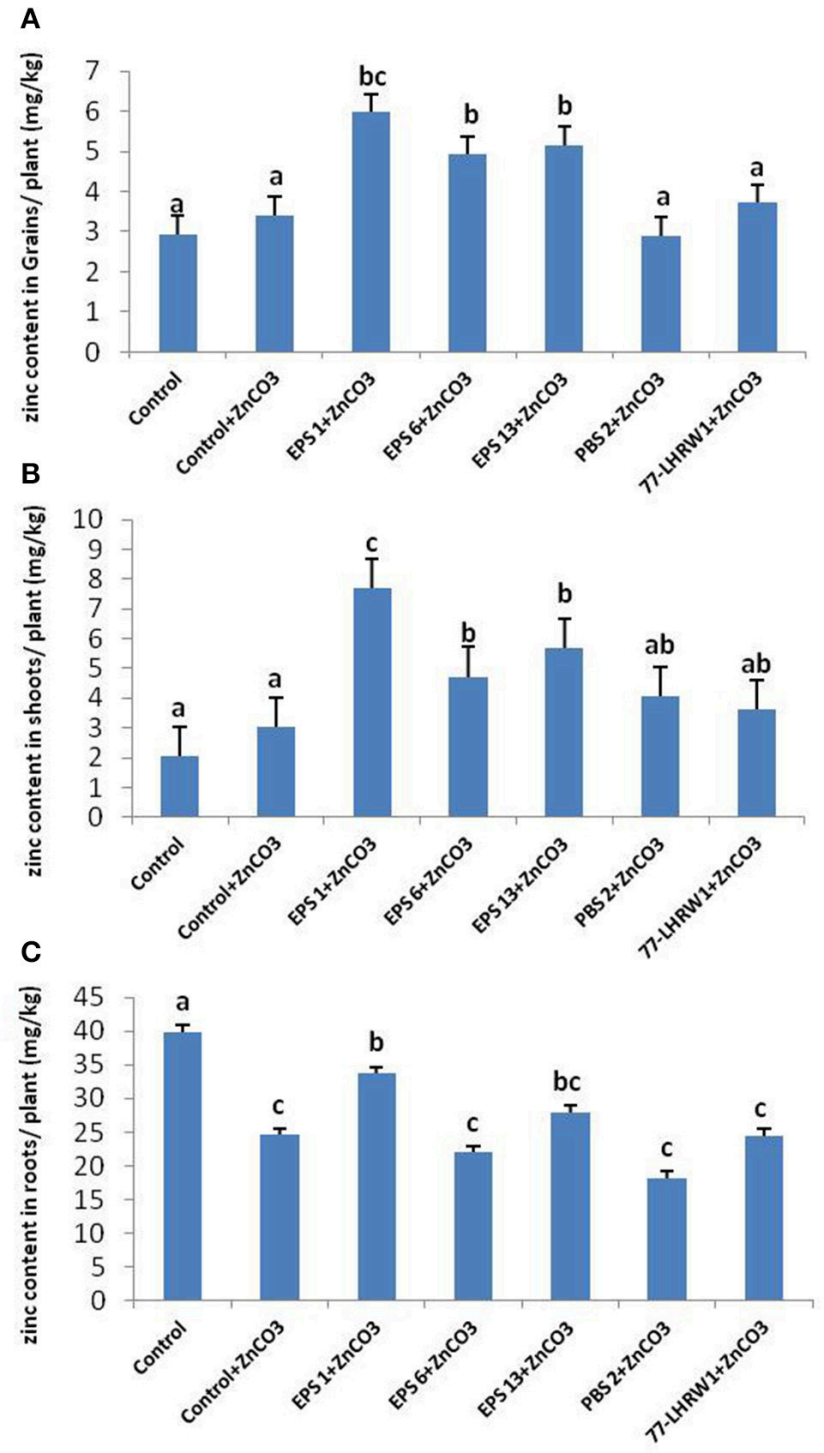
Quantification of Zinc content in **(A)** Grains, **(B)** Shoots, and **(C)** Roots of inoculated and un-inoculated plants. Alphabets (a–c) represent the significant and non-significant difference among datasets. ^a^Indicates no significant difference among dataset labeled with a on them. ^b^Shows a slight difference from (a) and same group category for dataset labeled with (b). ^c^Shows significant difference from a and b datasets and same data group labeled with c.

*Zinc content* Significant increase in zinc content of grains was observed for the plants inoculated with *Pseudomonas fragi* (EPS 1), *Pantoea dispersa* (EPS 6) and *Pantoea agglomerans* (EPS 13). Highest amounts of zinc were detected in the grains of *Pseudomonas fragi* (EPS 1) inoculated plants with 6.96 mg/kg of zinc followed by *Pantoea dispersa* (EPS 6) and *Pantoea agglomerans* (EPS 13) which had 6.3 and 4.64 mg/kg of zinc, respectively. Plants inoculated with strains *E. cloacae* (PBS 2) and *Rhizobium* sp. (LHRW1) showed insignificant increase in grain zinc content as compared to un-inoculated controls, i.e., 2.66 mg/kg and 2.68 mg/kg in control+ZnCO_3_ (Figure 5A). These results indicate the efficacy of three PGPR strains; *Pseudomonas fragi* (EPS 1), *Pantoea dispersa* (EPS 6) and *Pantoea agglomerans* (EPS 13) that they enhanced the bioavailability of zinc and mobilized it toward wheat grains.

Comparison of control and inoculated plants showed considerable increase in zinc content of shoots of inoculated ones. Highest zinc content in shoots was seen for the plants inoculated with strain *P. fragi* (EPS 1); 8.83 mg/kg followed by *P. agglomerans* (EPS 13) and *P. dispersa* (EPS 6) which showed zinc content of 5.73 and 4.46 mg/kg, respectively. Though, *E. cloacae* (PBS 2) and *Rhizobium* sp. (LHRW1) also showed increase in zinc content of shoots but it was non-significant (Figure 5B).

Interestingly, control plants showed significant difference in zinc content of roots as compared to all inoculated treatments and highest zinc content was recorded for control plants i.e., 47.97 mg/kg followed by *P. fragi* (EPS 1) that was 42.53 mg/kg. Minimum zinc content was seen for the roots of the plants inoculated with strain *E. cloacae* (PBS 2) that was 18.207 mg/kg. All other strains showed non-significant difference of zinc content in comparison to ZnCO_3_ supplemented control plants (Figure 5C).

## Discussion

Zinc is the key constituent of plants and is very crucial for their development. Zinc deficiency is the most common micronutrient deficiency in crops worldwide and results in substantial losses in crop yields. Use of zinc fertilizers may not be cost effective in alleviating zinc deficiency and increasing crop yield. In addition to this, zinc fertilizers are underutilized in many countries like Pakistan, despite the widespread occurrence of zinc-deficient soils. These zinc deficient soils hamper the growth of many plants including staple foods such as wheat, rice, corn and sugarcane. Wheat yield is critically affected by zinc deficiency and local studies have shown the use of zinc fertilizers to overcome this (Khan et al., 2009; Ahmad et al., 2012; Joy et al., 2017). But use of chemical fertilizers threatens public health and environment and also puts farmer's livelihood in jeopardy. Therefore, use of chemical fertilizers has declined in many countries and growers are returning to organic farming.

Rhizobacteria play a vital role in environmental cycling processes such as solubilization of metals into soluble forms that are suitable for plant uptake. These ions and metals significantly improve plant growth and nutrition. The main focus of this study was to identify and characterize the strains that have potential to be used as zinc-biofertilizers. Based on zinc solubilization, three EPS producing strains of wheat and two of sugarcane, were selected. One of these strains was identified as *Pseudomonas fragi* (EPS 1), two as *Pantoea dispersa* (EPS 6) and *Pantoea agglomerans* (EPS13), one sugarcane strain as *Enterobacter cloacae* (PBS 2), and one as *Rhizobium* sp. (LHRW1). These bacterial genera are known to colonize rhizosphere of wheat and sugarcane and increasing plant growth (Baig et al., 2012; Verma et al., 2015). Large body of literature describes the potential of these PGPR genera to increase plant growth and crop yield.

Various species of *Pseudomonas* have extensively been studied in relation to plant growth promotion. *P. fluorescens* has shown indole acetic acid production resulting in enhanced growth of onion (Reetha et al., 2014) as well as phosphate solubilization (Oteino et al., 2015). Zinc solubilization (Pawar et al., 2015), HCN production and biocontrol properties of *Pseudomonas* sp. have also been reported (Tank and Saraf, 2009). However, few reports are available for the beneficial effect of *P. fragi* strains on plants. Selvakumar et al. (2009) reported P solubilization, IAA and HCN production, enhanced germination rate, plant biomass and uptake of nutrients by wheat plants upon inoculation with *P. fragi* strain. However, reports of zinc solubilization by *P. fragi* were not found. Species belonging to *Pantoea* have been extensively studied and reported to have many plant growth promoting abilities. *Pantoea agglomerans* has been reported to solubilize phosphorus and produce IAA, siderophores as well as ammonia. On inoculation with *P. agglomerans*, jute plants have shown significant increase in plant height, weight, chlorophyll content and total carbohydrate content (Majumdar and Chakraborty, 2015). Plant growth promoting abilities of *Enterobacter* species have been reported extensively. *Enterobacter asburiae* has shown IAA, HCN, ammonia and siderophore production, as well as phosphate solubilization (Ahemad and Khan, 2010). *Rhizobium* sp. and *E. cloacae* have been reported to produce phytohormones like acetoin and other bioactive compounds. It has also shown to solubilize phosphate. Inoculation of *Pisum sativum*, with these strains resulted in a significant increase in growth of the plant (Khalifa et al., 2016). In this study too, *E. cloacae* (EPS 14) and *Pantoea agglomerans* (EPS 17) have also shown highest IAA production with 12.125 and 8.449 μg/ml. These genera have also previously been reported for producing high amounts of indole-3-acetic acid; 23.006 μg/ml (Mohite, 2013).

Our findings are consistent with previously reported literature and use of Zinc solubilizing *Pseudomonas fragi, Pantoea agglomerans, E. cloacae*, and *Rhizobium* sp. showed encouraging results. When inoculated with these strains, wheat plants showed enhanced shoot and root length and weight as well as zinc content. Most of the strains used in this study are not yet reported for zinc solubilizing ability and for their effect on wheat growth. Significant difference was seen in root and shoot zinc content with all inoculated plants as compared to un-inoculated controls. *Pseudomonas fragi* (EPS 1), *Pantoea dispersa* (EPS 6) and *Pantoea agglomerans* (EPS 13) showed promising results when the grain zinc content of wheat was analyzed after 3 months. *Pseudomonas fragi* (EPS 1) showed highest grain zinc content followed by *Pantoea agglomerans* (EPS 13) and *Pantoea dispersa* (EPS 6). Though *Rhizobium* sp. (LHRW1) and *E. cloacae* (PBS2) significantly increased biomass of the plant in all three experiments but highest root dry weight was observed by *Pseudomonas fragi* strain EPS 1. Plants harvested after 3 months, also showed the maximum amount of zinc in roots of un-inoculated control plants when compared with inoculated ones which is evidence that these PGPR genera have successfully helped the plant in zinc solubilization and uptake. It supports that inoculation with this strain accelerated the bioavailability of zinc to the roots of the plants and provided it with more solubilized zinc as compared to un-inoculated controls.

Increased zinc content of roots and shoots as compared to un-inoculated plants is supported by the previous reports where inoculation of plants with PGPR has resulted in increased yield, enhanced plant growth and improved nutrition and many effective strains have been formulated as biofertilizers in this regard. The PGPR strains identified so far mainly belong to genra *Pseudomonas, Ochrobacterum, Bacillus, Azospirillum, Azotobacter, Rhizobium, Stenotrophomonas, Serratia*, and *Enterobacteria* (Maleki et al., 2011). In addition to increase the overall yield of the plant, PGPR have extensively been reported in combating nutrient deficiencies of the plants and has been given attention to be used as biofertilizers. For example, Ramesh et al. (2014) has reported increased mobilization of zinc by zinc solubilizing *Bacillus aryabhattai* in wheat and soybean. Recent studies have also revealed 7–12% enhanced zinc translocation toward wheat grains by certain strains of *Serratia* sp., *Bacillus* sp. *Pseudomonas* sp. and many others as compared to chemical zinc supplementation to the plant (Lefèvre et al., 2014). In addition to increase biomass, zinc solubilizing bacteria used in this study, have also significantly enhanced zinc content of shoots and roots in comparison to un-inoculated plants which is a well documented phenomenon and has been explained in many earlier research studies. Previous studies have demonstrated that application of PGPR or PGPE has enhanced zinc translocation toward rice and wheat grains and this ability of rhizobacteria or plant growth promoting endophytes (PGPE) is related with their capacity of executing successful plant microbe interactions such as induction of physiological processes, mineralization and solubilization (Lucas et al., 2014; Wang et al., 2014). Plants apoplasts are known for offering different growth conditions and hence, many rhizosphere bacterial strains can be the effective endophytic colonizers of the plants (Khan et al., 2015). Bacterial genera used in this study have also been reported to colonize plant roots as endophytes and thus significantly solubilize minerals and available nutrients. Research studies have also shown that plants, when supplied with different nutrients as chemical fertilizers or through biofortification, triggered many physiological changes that helped them to take up the nutrients from the soil (Chattha et al., 2017).

In addition to this, all five strains used in plant experiments could solubilize phosphate and produced indole-3-acetic acid. IAA can also be a contributing factor in increasing plant growth and biomass. Though, no source of insoluble P was present in soil, but P solubilization is an important parameter in improving plant growth and cannot be neglected.

Zinc solubilization by PGPR is relatively a newer approach and most of the strains have not yet been tested for this activity. This study indicates the potential of *Pantoea, E. cloacae* and especially *Pseudomonas fragi* to be used as bio-fertilizer and overcome zinc deficiency in countries like Pakistan where zinc fertilizers are under-used and are not cost effective.

## Author contributions

SK: Conducted major experiments; IS: manuscript writing and data analysis; DB: molecular analysis; MR: conducted HPLC analysis; KM: provided the basic lab infrastructure, Dean for Research and Postgraduate Studies; SM: edited manuscript, guided in whole experiment plan.

### Conflict of interest statement

The authors declare that the research was conducted in the absence of any commercial or financial relationships that could be construed as a potential conflict of interest.
