# Supplementary material for: Contribution of Zinc Solubilizing Bacteria in Growth Promotion and Zinc Content of Wheat
Source: Front Microbiol. 2017 Dec 21;8:2593. doi: 10.3389/fmicb.2017.02593 (PMC5743011; doi:10.3389/fmicb.2017.02593)
Supplement: Supplementary file 1 [file Presentation1.pptx]

## Slide 1
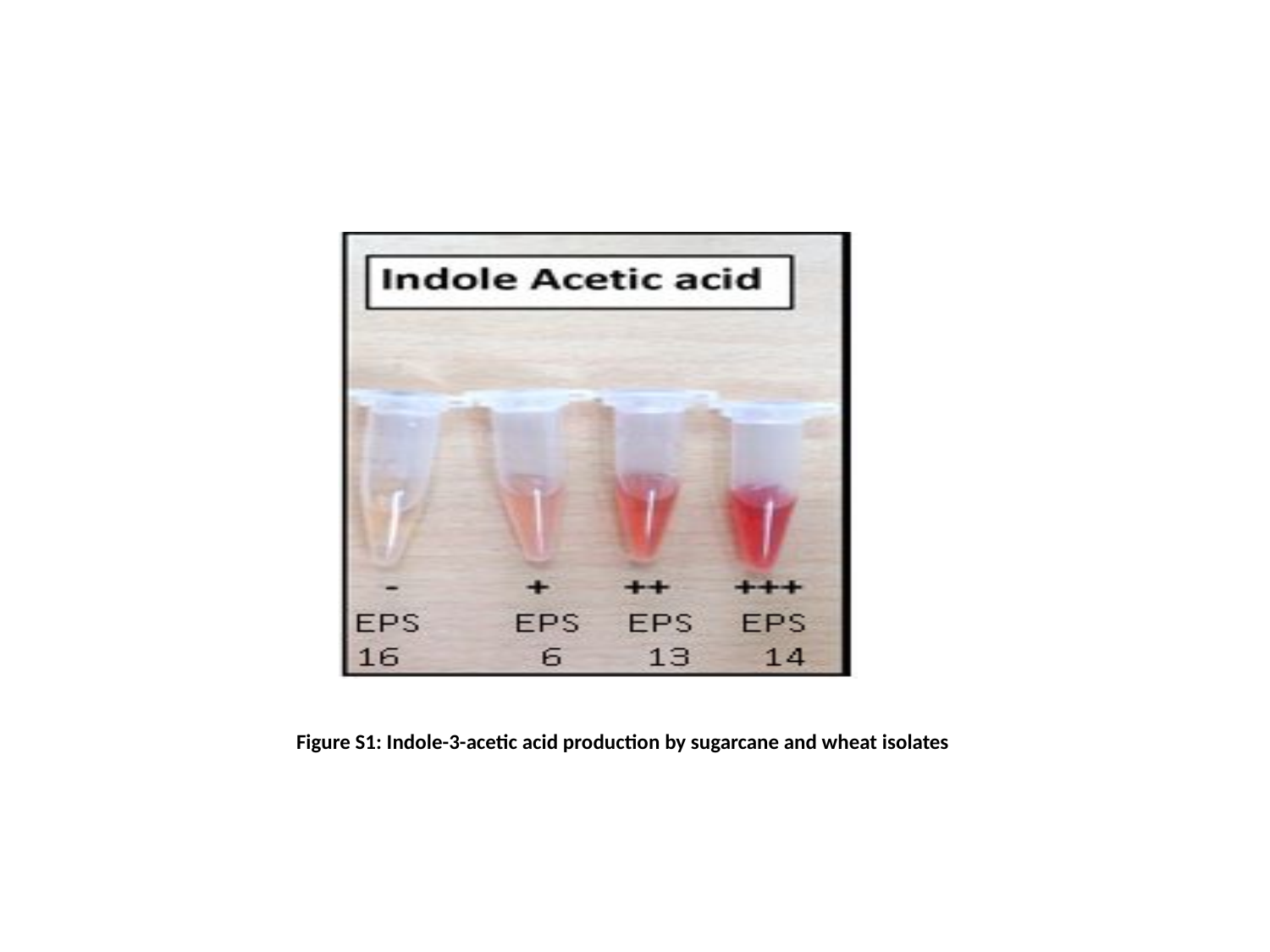

Figure S1: Indole-3-acetic acid production by sugarcane and wheat isolates

## Slide 2
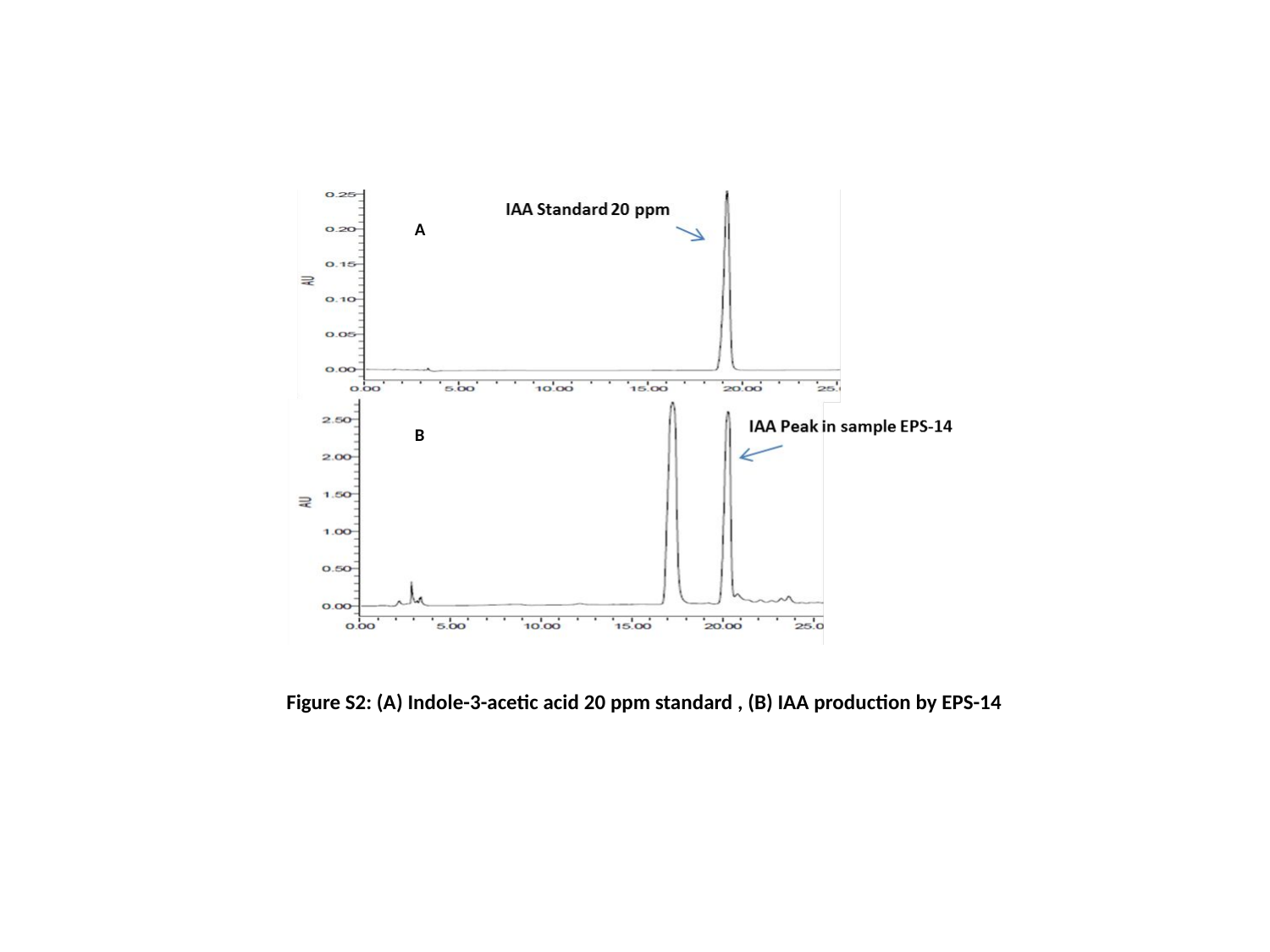

A
B
 Figure S2: (A) Indole-3-acetic acid 20 ppm standard , (B) IAA production by EPS-14

## Slide 3
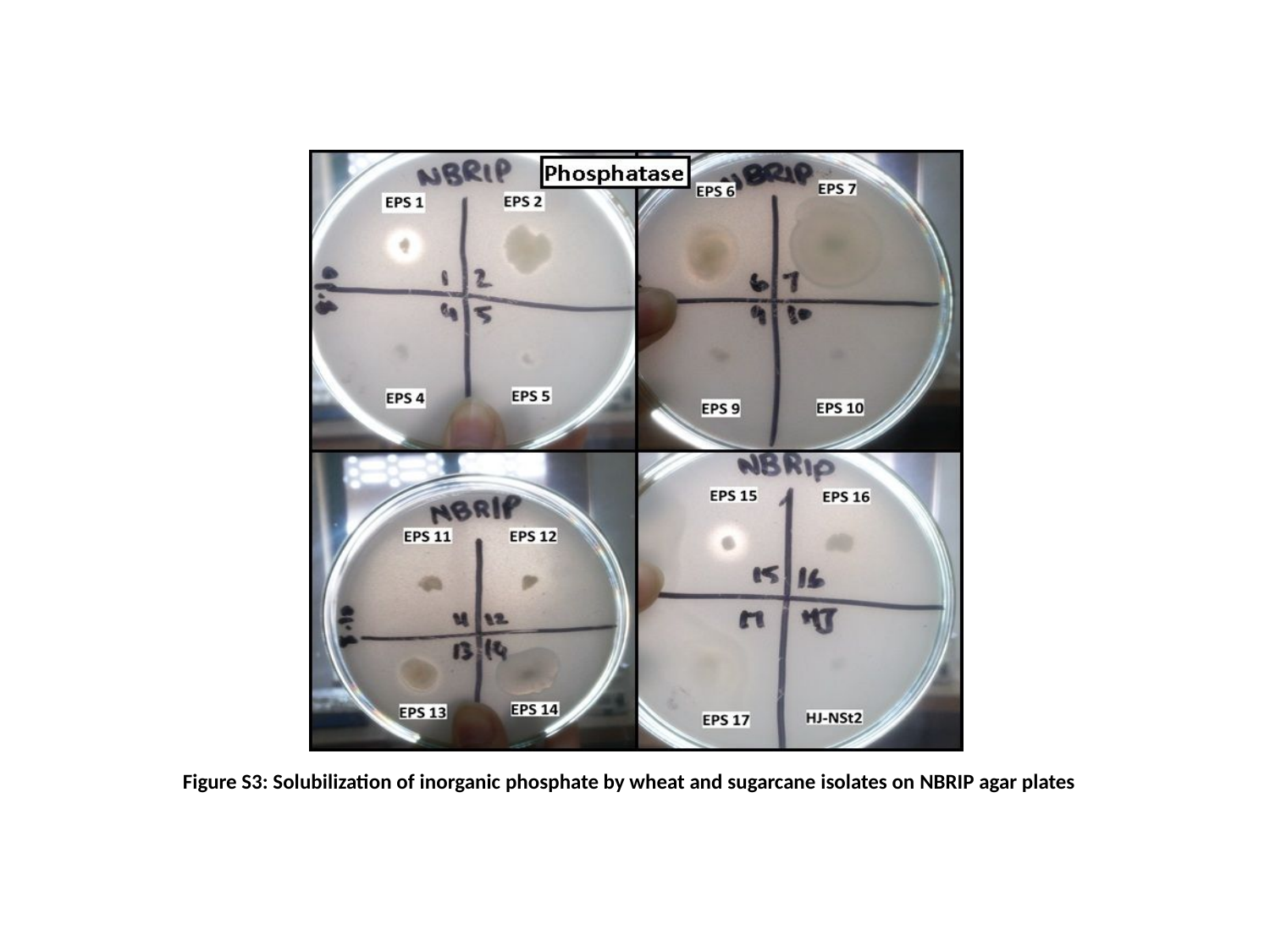

Figure S3: Solubilization of inorganic phosphate by wheat and sugarcane isolates on NBRIP agar plates

## Slide 4
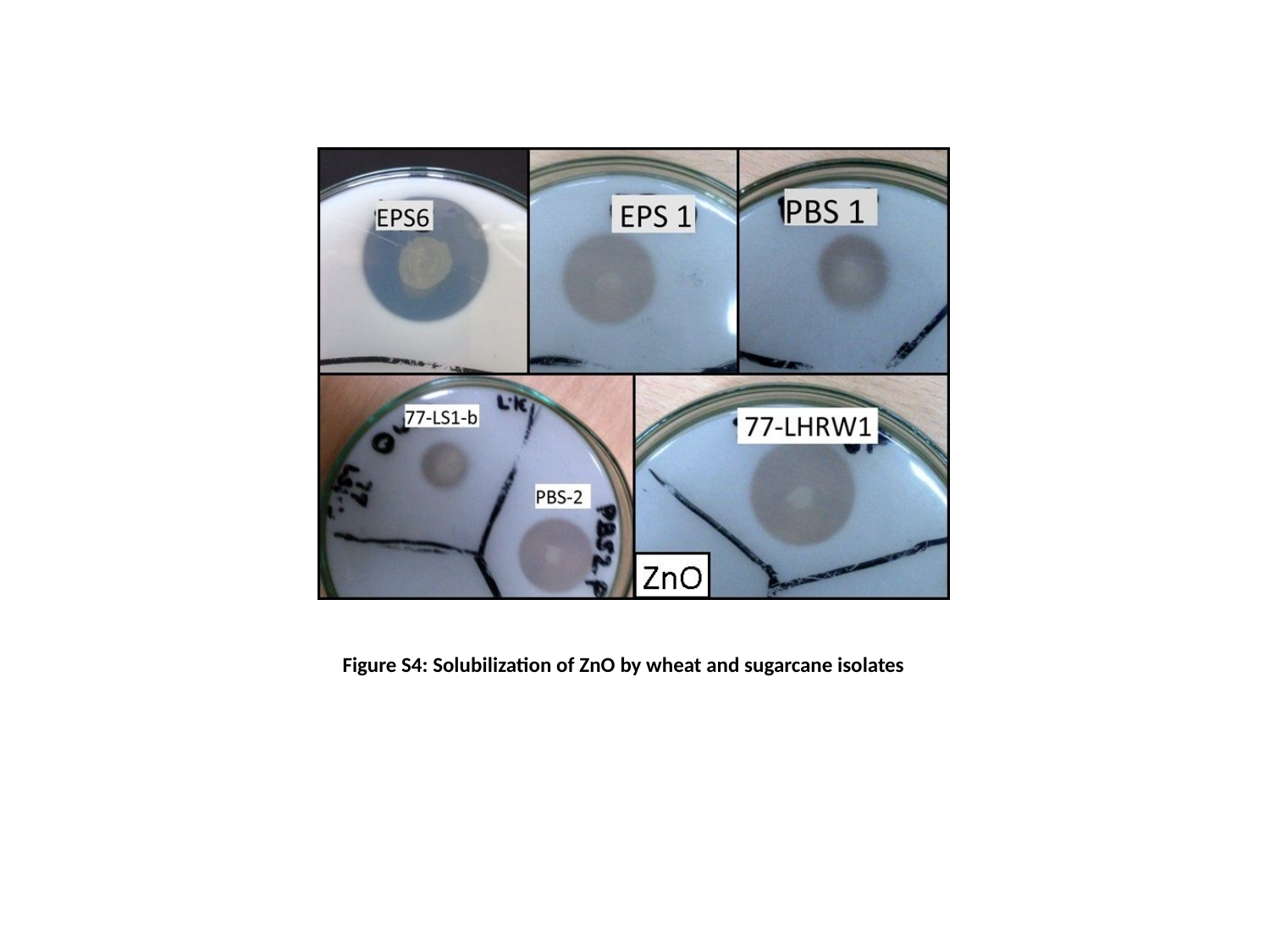

Figure S4: Solubilization of ZnO by wheat and sugarcane isolates

## Slide 5
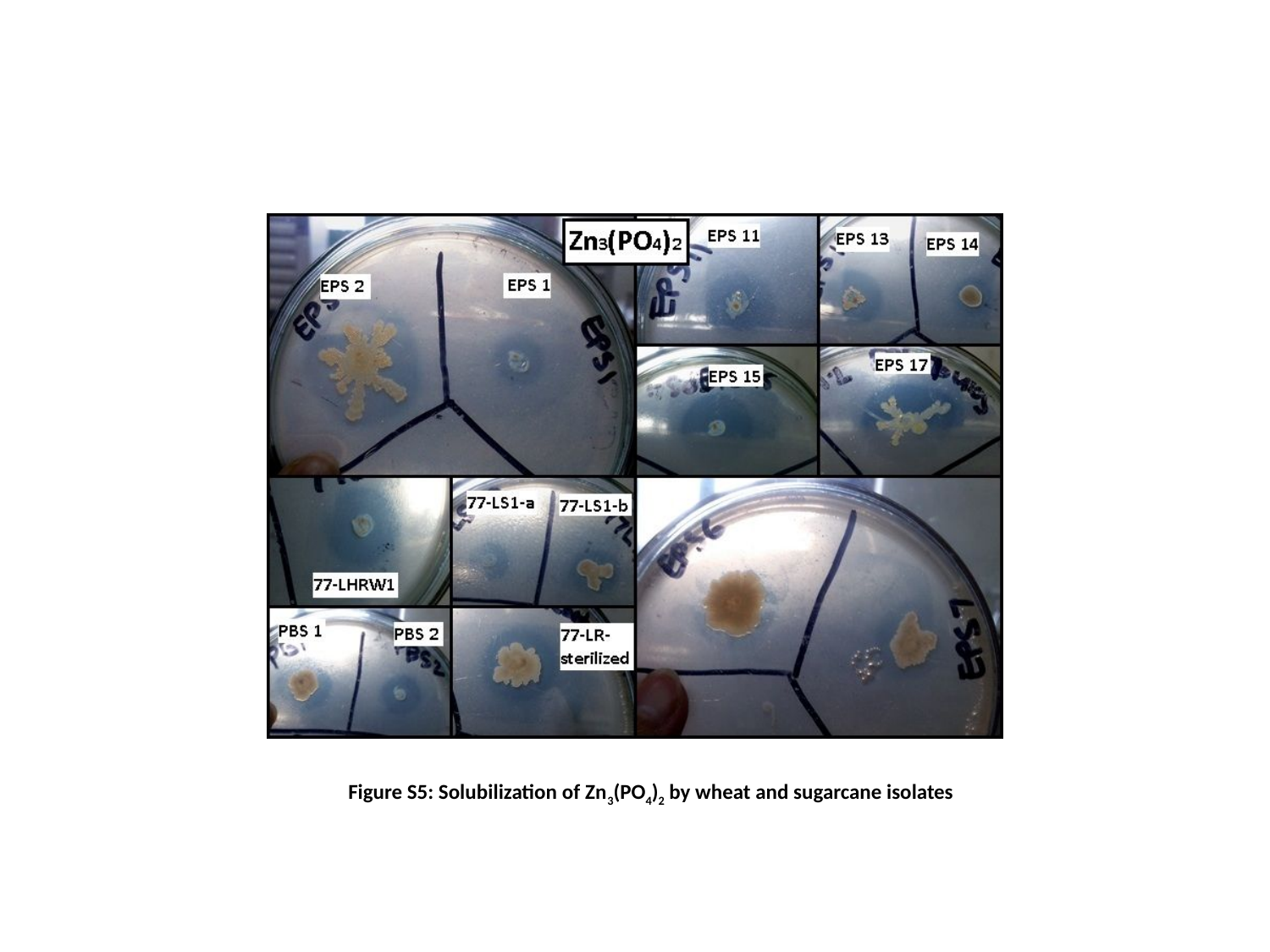

Figure S5: Solubilization of Zn3(PO4)2 by wheat and sugarcane isolates

## Slide 6
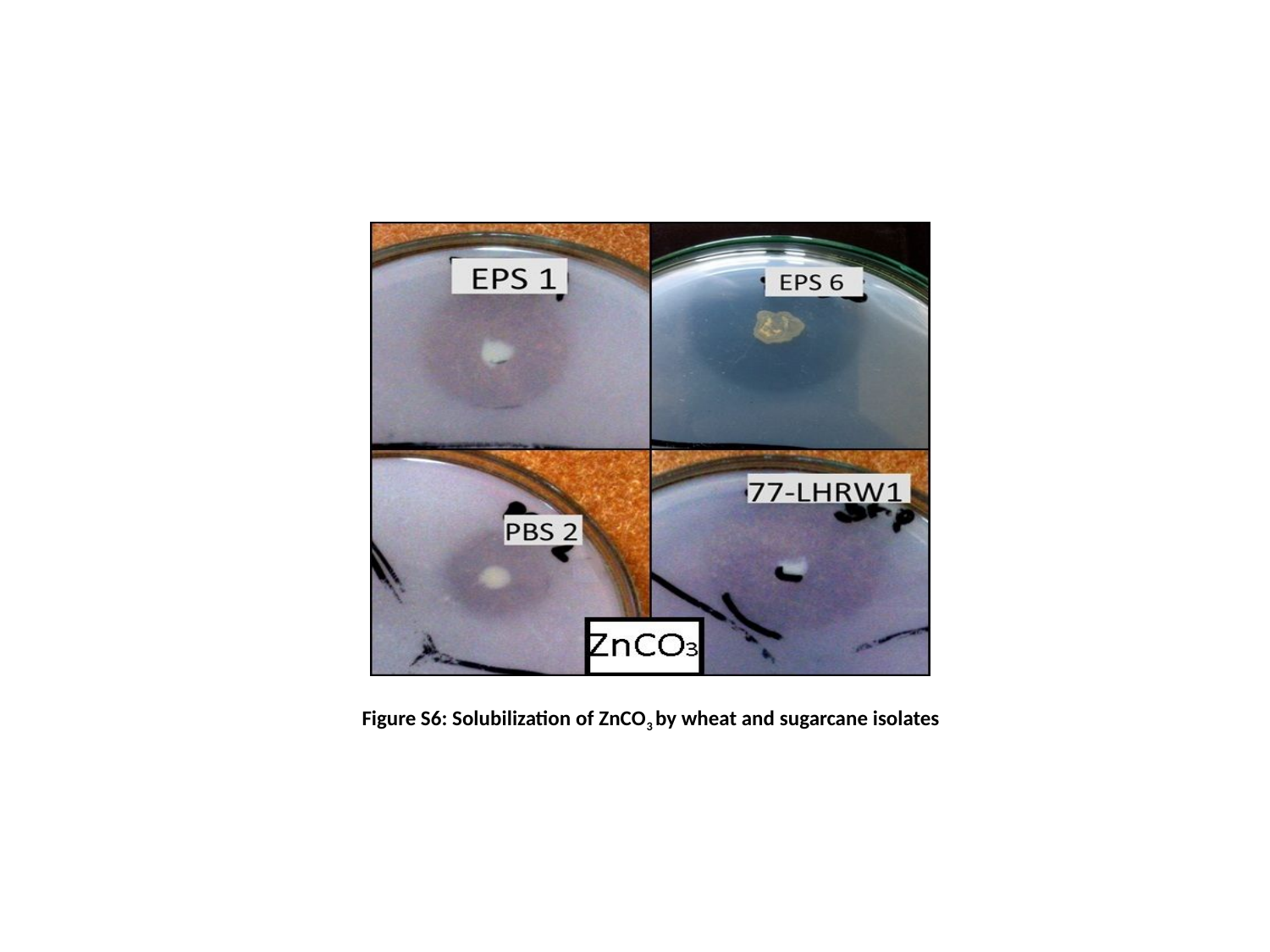

Figure S6: Solubilization of ZnCO3 by wheat and sugarcane isolates

## Slide 7
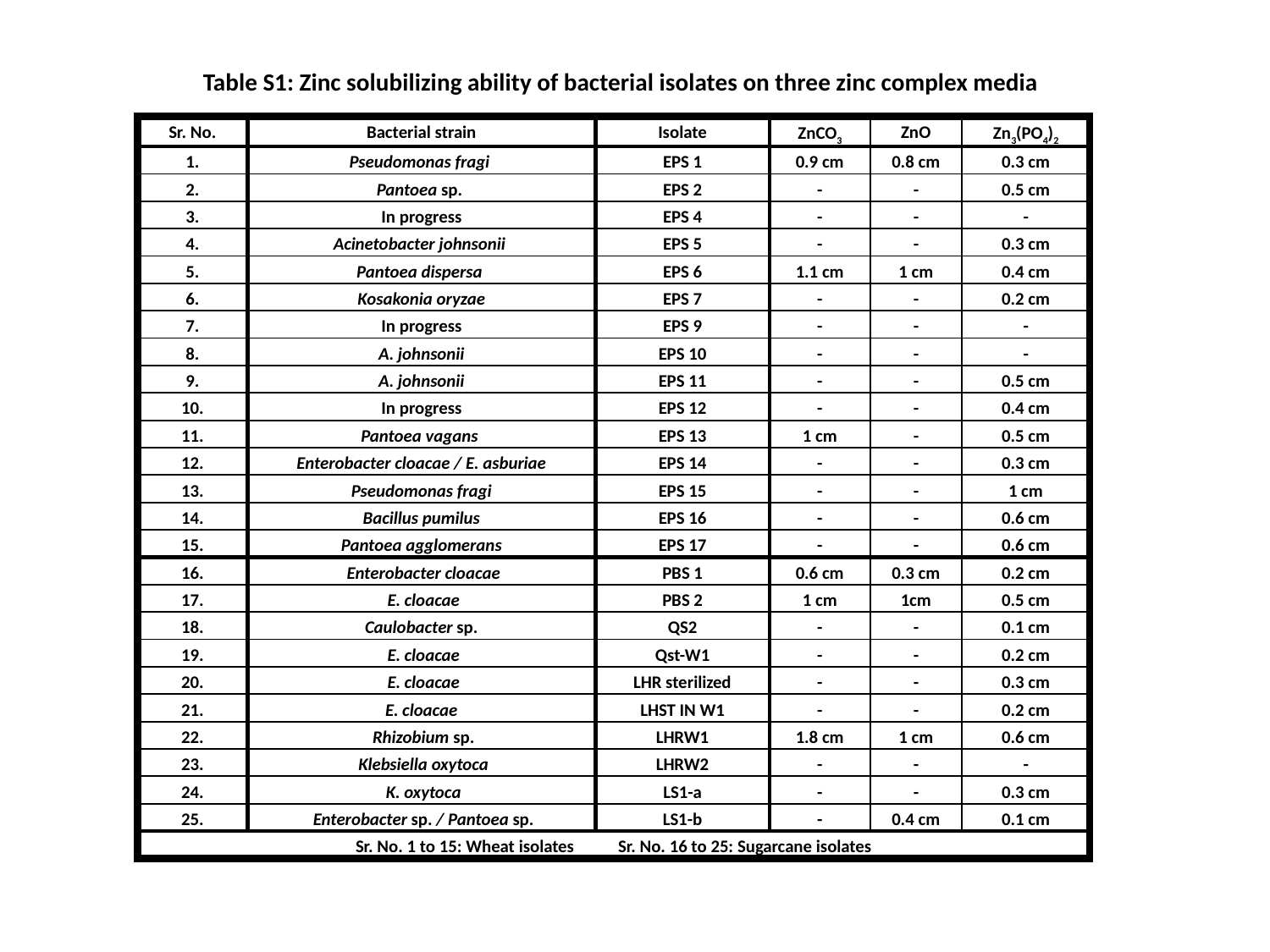

Table S1: Zinc solubilizing ability of bacterial isolates on three zinc complex media
| Sr. No. | Bacterial strain | Isolate | ZnCO3 | ZnO | Zn3(PO4)2 |
| --- | --- | --- | --- | --- | --- |
| 1. | Pseudomonas fragi | EPS 1 | 0.9 cm | 0.8 cm | 0.3 cm |
| 2. | Pantoea sp. | EPS 2 | - | - | 0.5 cm |
| 3. | In progress | EPS 4 | - | - | - |
| 4. | Acinetobacter johnsonii | EPS 5 | - | - | 0.3 cm |
| 5. | Pantoea dispersa | EPS 6 | 1.1 cm | 1 cm | 0.4 cm |
| 6. | Kosakonia oryzae | EPS 7 | - | - | 0.2 cm |
| 7. | In progress | EPS 9 | - | - | - |
| 8. | A. johnsonii | EPS 10 | - | - | - |
| 9. | A. johnsonii | EPS 11 | - | - | 0.5 cm |
| 10. | In progress | EPS 12 | - | - | 0.4 cm |
| 11. | Pantoea vagans | EPS 13 | 1 cm | - | 0.5 cm |
| 12. | Enterobacter cloacae / E. asburiae | EPS 14 | - | - | 0.3 cm |
| 13. | Pseudomonas fragi | EPS 15 | - | - | 1 cm |
| 14. | Bacillus pumilus | EPS 16 | - | - | 0.6 cm |
| 15. | Pantoea agglomerans | EPS 17 | - | - | 0.6 cm |
| 16. | Enterobacter cloacae | PBS 1 | 0.6 cm | 0.3 cm | 0.2 cm |
| 17. | E. cloacae | PBS 2 | 1 cm | 1cm | 0.5 cm |
| 18. | Caulobacter sp. | QS2 | - | - | 0.1 cm |
| 19. | E. cloacae | Qst-W1 | - | - | 0.2 cm |
| 20. | E. cloacae | LHR sterilized | - | - | 0.3 cm |
| 21. | E. cloacae | LHST IN W1 | - | - | 0.2 cm |
| 22. | Rhizobium sp. | LHRW1 | 1.8 cm | 1 cm | 0.6 cm |
| 23. | Klebsiella oxytoca | LHRW2 | - | - | - |
| 24. | K. oxytoca | LS1-a | - | - | 0.3 cm |
| 25. | Enterobacter sp. / Pantoea sp. | LS1-b | - | 0.4 cm | 0.1 cm |
| Sr. No. 1 to 15: Wheat isolates Sr. No. 16 to 25: Sugarcane isolates | | | | | |

## Slide 8
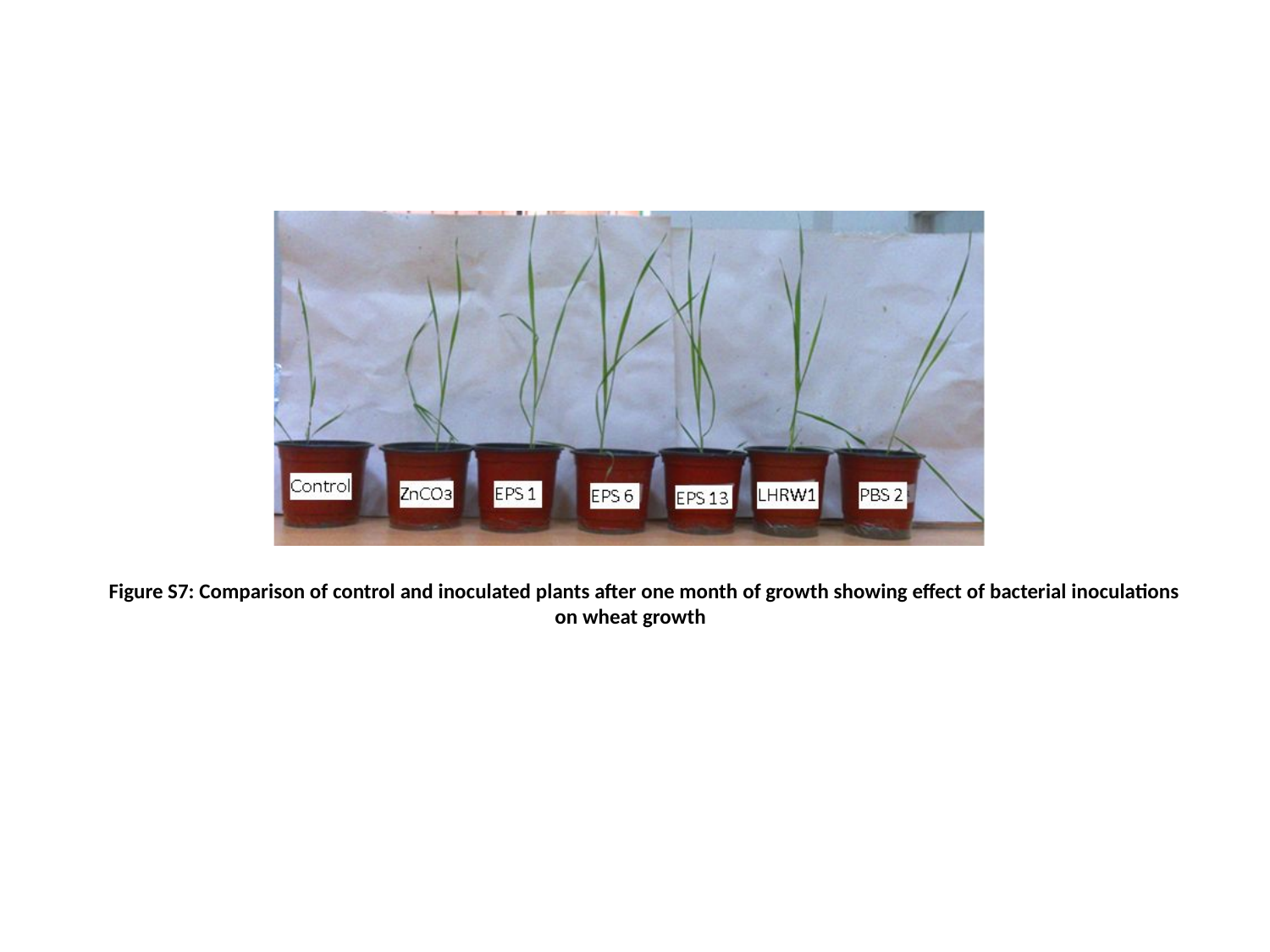

Figure S7: Comparison of control and inoculated plants after one month of growth showing effect of bacterial inoculations on wheat growth

## Slide 9
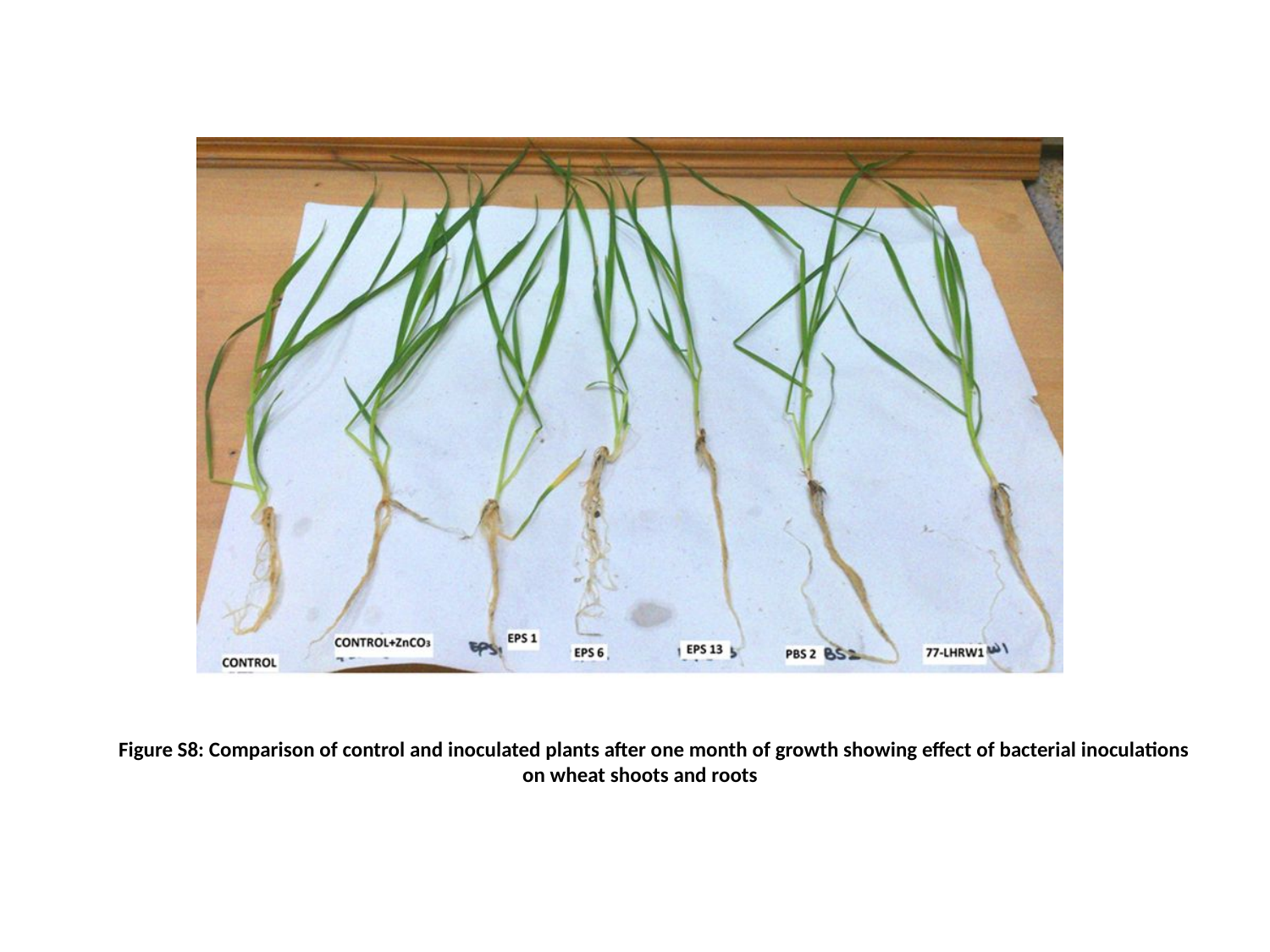

Figure S8: Comparison of control and inoculated plants after one month of growth showing effect of bacterial inoculations on wheat shoots and roots
